# Metachronous Bilateral Granulocytic Sarcoma of the Testis in a Young Adult: A Report of an Unusual Entity

**DOI:** 10.1155/2014/762630

**Published:** 2014-05-06

**Authors:** Fatih Hızlı, Hakan Aksüt, Aslı Mengeloğlu, İbrahim Sarı, Eşref Oğuz Güven, Halil Başar

**Affiliations:** ^1^Department of Urology, Oncology Training and Research Hospital, Demetevler, 06530 Ankara, Turkey; ^2^Department of Urology, Kilis State Hospital, Kilis, Turkey; ^3^Department of Pathology, Kilis State Hospital, Kilis, Turkey; ^4^Department of Pathology, Faculty of Medicine, Gaziantep University, Gaziantep, Turkey

## Abstract

Granulocytic sarcomas are rare tumors composed of neoplastic blood cells, typically occurring during the course of acute nonlymphoblastic leukemia or before its onset. We present a case of a 23-year-old young adult man with metachronous granulocytic sarcoma of the testis without hematologic manifestations who was diagnosed with granulocytic sarcoma (GS). The patient was treated with right orchiectomy but relapsed with a left testicular mass 16 months later when a left orchiectomy was performed. The patient has been free of disease for 13 months following the left orchiectomy. This case highlights a rare hematologic cancer that urologists and pathologists should be aware of since it can present as a testicular mass. Only 3 cases of testicular GS without an associated hematologic disorder have been described. To the best of our knowledge, our patient is the first reported case in the English literature of metachronous GS of the testis with no evidence of hematologic disorder.

## 1. Introduction

Granulocytic sarcomas are rare tumors composed of neoplastic blood cells, typically occurring during the course of acute nonlymphoblastic leukemia or before its onset. It infiltrates an extramedullary site and appears as a localized tumor mass. It has been reported in association with acute myeloid leukemia (AML), chronic leukemia, and other myeloproliferative disorders. Rarely, granulocytic sarcoma (GS) may be found in patients without hematologic evidence of leukemia or hematologic disorder, which is a diagnostic challenge, as in the case we report here.

In this paper, we present the first case in the English literature of metachronous GS of the testis.

## 2. Case Report

A 23-year-old young adult man had a painless right hemiscrotal mass for 5 to 6 months. He visited the urology clinic. On physical examination, a 2.5 × 1.5 cm nontender mass was found in the right testicle. The patient had no history of any major systemic disease. Laboratory data revealed normal serum levels of *α*-fetoprotein and *β*-human chorionic gonadotropin, and a systemic survey, which included chest radiography and abdominal computed tomography, could not identify any primary neoplasm or lymphadenopathy.

An intratesticular hypoechoic nodule was detected with the use of scrotal ultrasound. The lesion was approximately 2.5 × 1.5 × 1 cm in size and was located at the cranial aspect of the right testicle.

**Figure 1:**
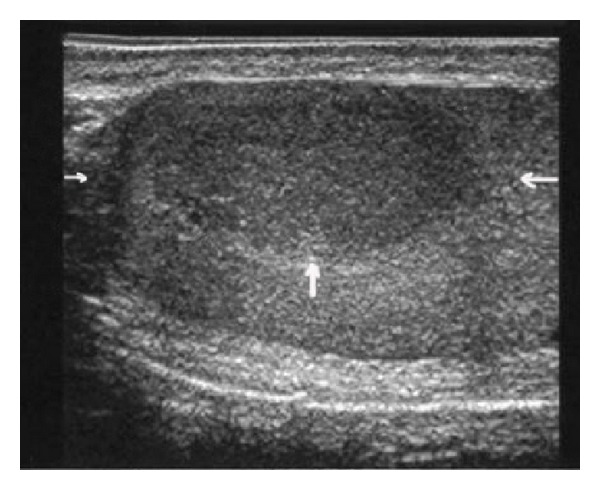
Scrotal sonogram showing a nodular lesion in right testicle (arrows). The lesion is homogeneously hypoechoic, well demarcated, and associated with distal enhancement.

It had a well-demarcated, smooth border and had a homogeneously hypoechoic echo pattern relative to the ipsilateral testis (Figure 1). The patient was treated with a right orchiectomy but relapsed 16 months later with a left testicular mass measuring about 5 × 4 cm in size, when a left orchiectomy was performed. As AML is a disease with diffuse bone marrow infiltration, we performed only one bone marrow biopsy, which was found to be free of disease. After right orchiectomy the patient was referred to the hematology department, but the patient refused systemic chemotherapy. Following relapse the patient consulted with a hematologist again. As he refused chemotherapy, he was followed up with left orchiectomy only.

The patient has been free of disease for 13 months following the left orchiectomy.

**Figure 2:**
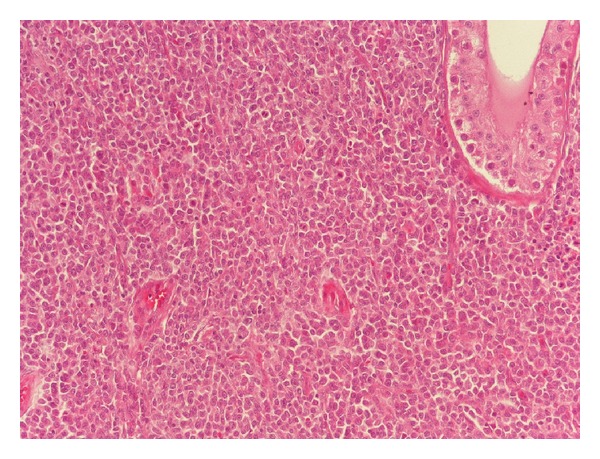
Histopathologic appearance of the granulocytic sarcoma. Granulocytic sarcoma de novo, testis. Multiple mitotic figures are seen (hematoxylin-eosin, ×200).

**Figure 3:**
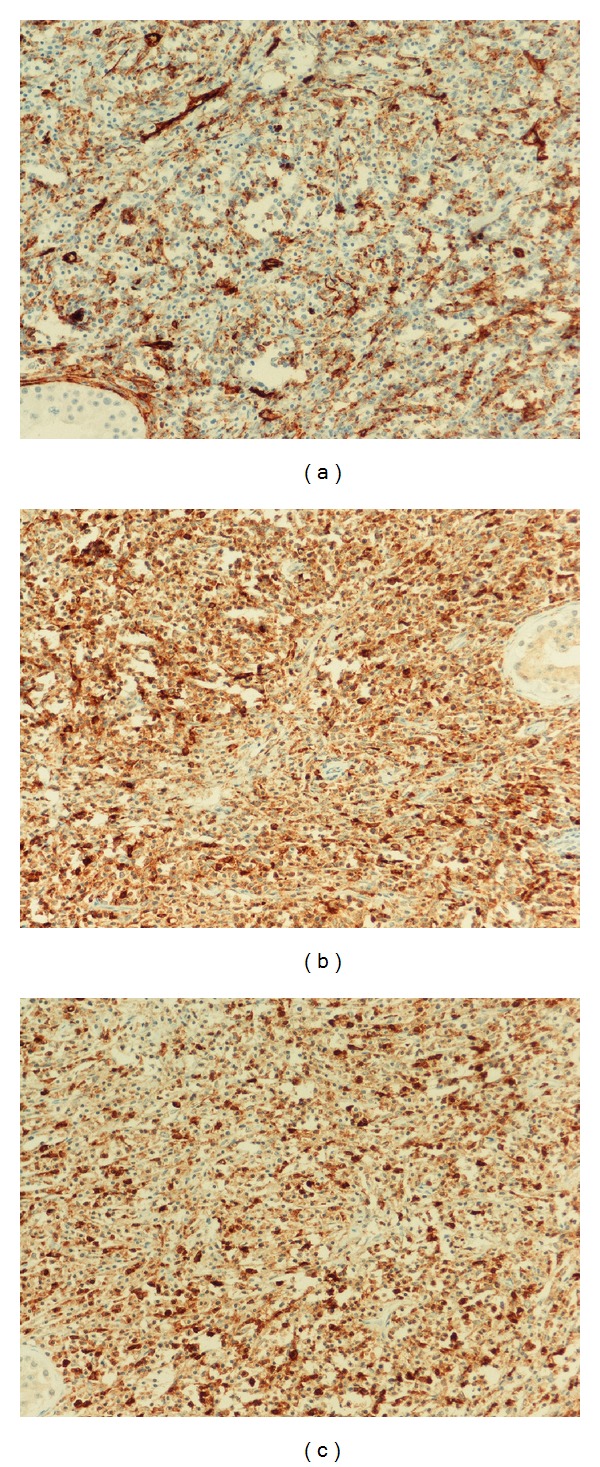
(a) Immunohistochemical findings: neoplastic cells are positive for CD34 (DAB, ×200). (b) Immunohistochemical findings: neoplastic cells are positive for CD68PGM1 (DAB, ×200). (c) Immunohistochemical findings: neoplastic cells are positive for MPO (DAB, ×200).

On gross examination, the lesion appeared as a white, firm mass. Microscopic examination revealed diffuse infiltration of myeloblastic, monoblastic cells (Figure 2). On immunohistochemistry in paraffin sections, CD34 (Figure 3), CD117, MPO, CD68KP1, CD68PGM1, and CD43 were expressed. The final diagnosis of the testicular tumor was GS.

**Figure 4:**
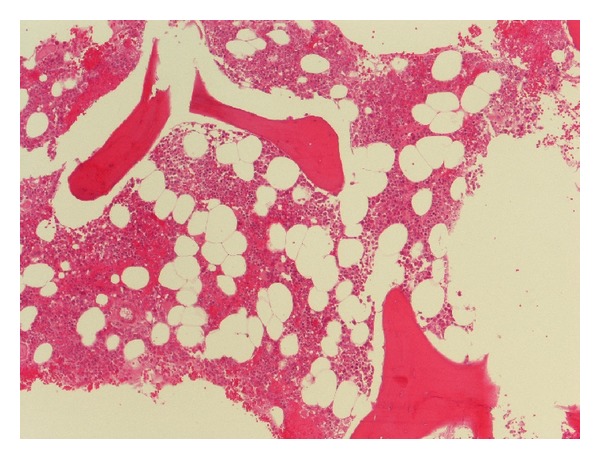
Bone marrow findings: no tumor cells were seen (hematoxylin-eosin, ×100).

A bone marrow biopsy was performed, which revealed normocellularity and no tumor (Figure 4). Currently, after a follow-up period of 13 months, the patient is still free of disease.

## 3. Discussion

Granulocytic sarcoma (GS), also known as chloroma, is a localized mass of primitive myeloid cells of the granulocytic series. The original name chloroma was first used by King [1] in 1853 because of the characteristic greenish color of typical tumors. The greenish color is produced by the enzyme myeloperoxidase. GS is most often found in combination with preexisting AML. It is also associated with other myeloproliferative and myelodysplastic disorders [2]. The prevalence has been estimated to be approximately 4.5% in chronic myelocytic leukemia [3] and 3.1% to 9.1% in AML [2].

There have been many case reports of GS in patients without leukemia in the literature. Most of the reports have suggested that GS almost invariably progresses to AML in patients without leukemia within a mean time span of 10 months [2], but this finding is still controversial. Acute myeloid leukemia did not develop in some patients with GS during the follow-up period [4].

The prevalence of testicular involvement in patients with lymphoproliferative disease is high, up to 64% in patients with acute leukemia and 25% in patients with chronic leukemia [5].

For a confirmative pathologic diagnosis of GS in the absence of leukemia, it is necessary to have light microscopic examination and immunohistochemical staining. The use of immunohistochemical stains such as those for myeloperoxidase, CD34, CD43, CD117, and lysozyme may identify up to 96% of extramedullary leukemia cases [6].

GS has been described as occurring at many body sites, such as the skin, intracranial and pineal regions, orbit, mandible, soft tissue, epidural region, breast, liver, pancreas, stomach, small intestine, ovary, uterus, prostate, urinary bladder, and testis. The most common sites are the cranium, facial bones, subcutaneous tissue, and lymph nodes. Involvement of multiple sites has been reported, and tumors may recur at their original locations [7, 8].

Economopoulos et al. [9] reported a case of primary testicular GS. The 44-year-old patient had a painless left testicular mass interpreted pathologically as “myelosarcoma.” Iliac and paraaortic lymph node involvement was shown at staging laparotomy. Fifteen months after a left orchiectomy, the patient had a relapse with a right testicular mass. A right orchiectomy was performed, and the pathologic diagnosis was GS. Myelogenous leukemia developed 24 months after the first episode of the left testicular mass. The authors concluded that the primary GS represented a systemic disease.

The patient in our case was treated with a right orchiectomy but relapsed 16 months later with a left testicular mass measuring about 5 × 4 cm in size, when a left orchiectomy was performed. The patient has been free of disease for 13 months following the left orchiectomy.

This case highlights a rare hematologic cancer that urologists and pathologists should be aware of since it can present as a testicular mass. Only 3 cases of testicular GS without an associated hematologic disorder have been described in the literature [10]. To the best of our knowledge, our patient is the first reported case in the English literature of metachronous GS of the testis with no evidence of hematologic disorder.
